# Generation of myogenic progenitor cell-derived smooth muscle cells for sphincter regeneration

**DOI:** 10.1186/s13287-020-01749-w

**Published:** 2020-06-12

**Authors:** Marco Thurner, Martin Deutsch, Katrin Janke, Franka Messner, Christina Kreutzer, Stanislav Beyl, Sébastien Couillard-Després, Steffen Hering, Jakob Troppmair, Rainer Marksteiner

**Affiliations:** 1 Innovacell Biotechnologie AG, Mitterweg 24, 6020 Innsbruck, Austria; 2 Daniel Swarovski Research Laboratory (DSL), Visceral Transplant and Thoracic Surgery, Medical University of Innsbruck, Innsbruck, Austria; 3 Institute of Experimental Neuroregeneration, Spinal Cord Injury and Tissue Regeneration Center Salzburg (SCI-TReCS), Paracelsus Medical University, Salzburg, Austria; 4 Austrian Cluster for Tissue Regeneration, Vienna, Austria; 5 Department of Pharmacology and Toxicology, University of Vienna, Vienna, Austria

**Keywords:** Skeletal muscle-derived cells, Smooth muscle, Myogenic progenitor cells, Satellite cells, Smooth muscle regeneration, Cell therapy, Regenerative medicine, Sphincter regeneration, Fecal incontinence, Tissue engineering

## Abstract

**Background:**

Degeneration of smooth muscles in sphincters can cause debilitating diseases such as fecal incontinence. Skeletal muscle-derived cells have been effectively used in clinics for the regeneration of the skeletal muscle sphincters, such as the external anal or urinary sphincter. However, little is known about the in vitro smooth muscle differentiation potential and in vivo regenerative potential of skeletal muscle-derived cells.

**Methods:**

Myogenic progenitor cells (MPC) were isolated from the skeletal muscle and analyzed by flow cytometry and in vitro differentiation assays. The differentiation of MPC to smooth muscle cells (MPC-SMC) was evaluated by immunofluorescence, flow cytometry, patch-clamp, collagen contraction, and microarray gene expression analysis. In vivo engraftment of MPC-SMC was monitored by transplanting reporter protein-expressing cells into the pyloric sphincter of immunodeficient mice.

**Results:**

MPC derived from human skeletal muscle expressed mesenchymal surface markers and exhibit skeletal myogenic differentiation potential in vitro. In contrast, they lack hematopoietic surface marker, as well as adipogenic, osteogenic, and chondrogenic differentiation potential in vitro*.* Cultivation of MPC in smooth muscle differentiation medium significantly increases the fraction of alpha smooth muscle actin (aSMA) and smoothelin-positive cells, while leaving the number of desmin-positive cells unchanged. Smooth muscle-differentiated MPC (MPC-SMC) exhibit increased expression of smooth muscle-related genes, significantly enhanced numbers of CD146- and CD49a-positive cells, and in vitro contractility and express functional Ca_v_ and K_v_ channels. MPC to MPC-SMC differentiation was also accompanied by a reduction in their skeletal muscle differentiation potential. Upon removal of the smooth muscle differentiation medium, a major fraction of MPC-SMC remained positive for aSMA, suggesting the definitive acquisition of their phenotype. Transplantation of murine MPC-SMC into the mouse pyloric sphincter revealed engraftment of MPC-SMC based on aSMA protein expression within the host smooth muscle tissue.

**Conclusions:**

Our work confirms the ability of MPC to give rise to smooth muscle cells (MPC-SMC) with a well-defined and stable phenotype. Moreover, the engraftment of in vitro-differentiated murine MPC-SMC into the pyloric sphincter in vivo underscores the potential of this cell population as a novel cell therapeutic treatment for smooth muscle regeneration of sphincters.

## Background

Sphincters are circular muscles controlling the movement of solids and/or liquids. They consist of either skeletal muscles, such as the external anal sphincter or smooth muscles, such as the internal anal and pyloric sphincters [1, 2]. Malfunction of the sphincter muscles of the anus and the pylorus is associated with fecal incontinence and gastroparesis, respectively [3, 4]. Although not life-threatening, fecal incontinence severely affects patients’ quality of life [5]. The prevalence rate of fecal incontinence is estimated to be up to 12% in men and women [6, 7], and the main type, with approximately 78% of all cases [8], results from the degeneration of the smooth muscle of the internal anal sphincter which causes passive fecal incontinence [9]. Conservative treatments such as the application of bulking agents have limited success in patients with high incontinence severity, and surgical approaches have high morbidity and complication rates [10]. Thus, there is a strong need for cell therapeutic approaches for the regeneration of smooth muscle tissue building sphincters.

The functionality of smooth muscle relies on highly differentiated smooth muscle cells expressing contractile proteins, such as alpha smooth muscle actin (aSMA), desmin, and smoothelin (SMTN) [11–13], as well as functional voltage-gated calcium and potassium channels, enabling regulated cell contraction [14].

Treatment of multipotent mesenchymal stromal cells (MSC) with transforming growth factor beta (TGFb) has been shown to induce the expression of smooth muscle-specific genes under the regulation of the CArG box via the signaling through SMADs, serum response factor, and myocardin [15–17]. Thus, using MSC of various origins, the application of TGFb1 during differentiation was shown to upregulate the expression of smooth muscle-specific contractile proteins [18] and functional calcium and potassium channels [19].

Skeletal muscle tissue is a source of stem and progenitor cells expected to be endowed with high regenerative potential, such as MSC and satellite cell-derived myogenic progenitor cells (MPC) [20]. Skeletal muscle-derived CD56^+^ cells have been shown to improve patients’ external anal sphincter-associated fecal incontinence in clinics [21–26]. Furthermore, skeletal muscle-derived cells were found to engraft into the bladder detrusor muscle improving bladder function [27]. However, limited knowledge exists regarding the differentiation potential and regenerative capacity of skeletal muscle-derived cells for the treatment of smooth muscle-related deficiencies [28], particularly for sphincter smooth muscle regeneration.

In the study presented here, we generated in vitro smooth muscle cells from skeletal muscle-derived myogenic progenitor cells and evaluated their cell therapeutic potential for sphincter regeneration.

## Methods

### Isolation and culture of human skeletal muscle-derived cells

Muscle samples of human origin were retrieved as a part of a clinical trial (EudraCT Number: 2010-021463-32), and residual material of patients agreeing to further usage of their cells was used for research purposes following informed consent. Cells were isolated from muscle biopsies (*Musculus pectoralis major* or *Latissimus dorsi*) and expanded under cGMP environment as described before [29, 30]. Cells were maintained by standard cell culture methods. Briefly, cells were cultured in a growth medium containing Ham’s F-10 basal medium supplemented with 10% FCS (inactivated at 57 °C, 40 min, Life Technologies, UK), bFGF (CellGenix, Freiburg, Germany), and gentamicin (Sandoz GmbH, Austria) and incubated at 37 °C, 5% CO_2_. Counting of cells was performed on Nucleocounter™ (ChemoMetec, Allerod, Denmark) according to the manufacturer’s instructions.

### Isolation, culture, and transplantation of murine skeletal muscle-derived cells

Murine skeletal muscle-derived cells were obtained from skeletal muscle biopsies of Gt (ROSA)26Sortm4(ACTB-tdTomato,-EGFP)Luo/J, in short, TdTomato mice (Jackson Laboratory, ME, USA). Adult mice were sacrificed by cervical dislocation. Next, samples were obtained from the *longissimus dorsi*, *gastrocnemius*, and *tibialis anterior* muscles using scissors and scalpel. The muscles were transferred into a sterile petri dish and covered with 1× PBS. Then, using tweezers and a scalpel, the remaining connective tissue was removed from the skeletal muscle and discarded. Afterwards, the muscle tissue was digested using the skeletal muscle dissociation kit (MiltenyiBiotec GmbH, Bergisch Gladbach, Germany) following the manufacturer’s instructions. In order to separate myogenic progenitor cells (mMPC) from non-myogenic cells, a satellite cell isolation kit (Miltenyi Biotec, Bergisch Gladbach, Germany) was used according to the manufacturer’s instructions. Collected mMPC and non-myogenic cells were centrifuged for 10 min at 400×*g* and resuspended in mouse growth medium, consisting of DMEM/Ham’s F12 supplemented with 20% FCS and bFGF. Murine cells were cultivated on collagen-coated culture flasks, prepared by covering the surface of the culture flasks with collagen I from the rat tail (Sigma-Aldrich Co. LLC, MO, USA) diluted 1:10 1× PBS for 1 h at 37 °C. Subcultivation, cryopreservation, cell count, and immunostainings were performed like for human skeletal muscle-derived cells.

### Separation of MPC and MSC by MACS

MSC (CD56^−^ skeletal muscle-derived cells) were separated from MPC (CD56^+^ skeletal muscle-derived cells) by magnetic-activated cell sorting (MACS). The human CD56 MicroBeads kit (MiltenyiBiotec GmbH, Bergisch Gladbach, Germany) was used for the separation of CD56^+^ and CD56^−^ cells according to the manufacturer’s instructions.

### Human bladder smooth muscle cells

Primary human bladder smooth muscle cells (HBdSMC-c) were obtained from PromoCell® (Cat. No.: C-12571) and cultivated in growth medium (Ham’s F10, 10% FCS, bFGF, gentamicin) for expansion to reach synthetic smooth muscle cells (s-hBd-SMC). Phenotype switch to contractile smooth muscle cells (hBd-SMC) was induced by switching to smooth muscle differentiation medium and cultivating cells therein for 6 days.

### Flow cytometry

Flow cytometry analysis was performed on a Guava easyCyte 6HT 2 L flow cytometer (Merck Millipore, Darmstadt, Germany) as described before [29]. Before measurement, 40,000 cells were resuspended in 195 μl 1× PBS and incubated after addition of 5 μl CD34-PE, CD56-PE, CD146-PE, IgG1-PE, IgG1-FITC, CD90-PE, CD105-PE, HLA-DR-PE, CD45-PE (all from Beckman Coulter, CA, USA), CD49a-FITC (MiltenyiBiotec GmbH, Bergisch Gladbach, Germany), CD14-PE (Thermo Scientific, MA, USA), CD19-PE (BioLegend, CA, USA), or CD73-PE (Becton Dickinson, NJ, USA) for 30 min at 4 °C in the dark following a washing step with 1× PBS. Histograms and dot plots were generated with a minimum of 5000 events at a sample flow rate of 1.8 μl/ml. Positive staining was obtained by comparison with isotype control set as 99% negative or comparison to control (negative) cells.

### Immunofluorescence staining on cell cultures

Immunofluorescence staining was performed directly on gelatine-coated 24-well plates or glass coverslips placed in 6-well plates as described before [29]. For fluorescent immunolabeling of alpha smooth muscle actin (aSMA), smoothelin, smooth muscle myosin heavy chain (SM-MHC), or desmin, cells were incubated with a mouse anti-actin alpha smooth muscle (Sigma-Aldrich Co. LLC, MO, USA), mouse anti-smoothelin (Merck Millipore, MA, USA), anti-smooth muscle myosin heavy chain (Merck Millipore, MA, USA), or rabbit anti-desmin (Thermo Scientific, MA, USA) antibody, respectively, each diluted 1:100 in blocking medium. Secondary goat anti-mouse Alexa488 or donkey anti-rabbit Alexa547-conjugated antibodies (Thermo Scientific, MA, USA), diluted 1:200 in blocking medium, were used. Counterstaining of the nuclei was performed by incubating the cells with Hoechst33342 (Sigma-Aldrich Co. LLC, MO, USA) diluted to a final concentration of 2 μg/ml in PBST (0.1% Triton X-100). Cells were mounted with Entellan® (Merck Millipore, MA, USA) and sealed with glass coverslips. Stainings were compared to procedures without primary antibodies and cells negative for tested antibodies. Double staining procedures were performed by simultaneous incubation with mouse anti-aSMA or mouse anti-smoothelin and rabbit anti-desmin primary antibodies. This was followed by incubation with combinations of secondary antibodies: goat anti-mouse Alexa488 and goat anti-rabbit Alexa547. Double stain procedures were controlled for unspecific binding of secondary antibodies by omitting one first antibody demonstrating the absence of cross staining with the respective other secondary antibodies.

### Skeletal muscle differentiation

Skeletal muscle-derived cells were differentiated in 24-well Nunclon™ Delta Surface plastic plates (Thermo Scientific, MA, USA) by replacing the growth medium with Skeletal Muscle Cell Differentiation Medium (500 ml, PromoCell GmbH, Germany), supplemented with 10 ml of Skeletal Muscle Cell Differentiation Medium Supplement Pack (PromoCell GmbH, Germany) and 240 μl gentamicin (8 mg/ml, Sandoz GmbH, Austria) as described before [29].

### Adipogenic, chondrogenic, and osteogenic differentiation

For adipogenic, chondrogenic, and osteogenic differentiation in vitro, each 500,000 cells were seeded onto 6-well plates (NUNC, Thermo Scientific, MA, USA) and cultivated in a growth medium for 24 h at 37 °C, 5% CO_2_. Next, cells were washed once with 5 ml DMEM/Ham’s F12 and covered with each 5 ml adipogenic, chondrogenic, or osteogenic differentiation medium. Adipogenic, chondrogenic, and osteogenic differentiation medium consisted of StemXVivo™ Osteogenic/Adipogenic Base Medium (R&D Systems Inc., MN, USA), supplemented with StemXVivo Human/Mouse/Rat Adipogenic (R&D Systems Inc., MN, USA), StemXVivo Human Osteogenic Supplement (R&D Systems Inc., MN, USA), or STEMPro® Chondrogenesis Supplement (Gibco®, Thermo Scientific, MA, USA), respectively, according to the manufacturer’s instructions. Differentiation media were further supplemented with gentamicin (Sandoz GmbH, Austria) to reach a final concentration of 3.83 μg/ml. Cells were cultivated each for 14 days in respective differentiation media, which were changed every 2–3 days. After 14 days of cultivation, successful differentiation was assessed by the presence of adipocytes, chondrocytes, and osteocytes visualized by oil red o, alcian blue, and alizarin red s staining, respectively. Quantification of oil red o, alcian blue, and alizarin red s staining was performed on microscopic images of multiple individual experiments by ImageJ software package. Therefore, images were loaded and color channels split. Red channels were used for oil red o and alizarin red s stainings, while blue channel was used for alcian blue stainings. The background was eliminated by setting a common threshold, and the average pixel intensity per field was acquired for quantification by ImageJ.

### Smooth muscle differentiation

To induce smooth muscle differentiation of MPC, murine MPC and MSC to MPC-SMC, and murine MPC-SMC and MSC-SMC, cells cultivated with growth medium to a confluency of about 70% were washed once with DMEM/F12 (Thermo Scientific, MA, USA). Next, cells were covered with smooth muscle differentiation medium, consisting of DMEM/F12 supplemented with recombinant human TGFb1 (Thermo Scientific, MA, USA), heparin sodium salt from the porcine intestinal mucosa (Sigma-Aldrich Co. LLC, MO, USA), fetal calf serum (Gibco, Thermo Scientific, MA, USA), and gentamicin (Sandoz GmbH, Tirol, Austria) to a final concentration of 10 ng/ml, 5% (v/v), and 3.84 μg/ml, respectively. Finally, cells were cultivated in smooth muscle differentiation medium for 3–6 days at 37 °C, 5% CO_2_. The medium was changed every 3–4 days.

### Fusion index calculation

Fusion index (FI) calculation was performed as described before [29]. Fusion index was calculated for each captured field of view by dividing the number of nuclei within the tubes with the total number of nuclei per field, followed by the calculation of the mean for all analyzed fields. Only cells that have at least 3 nuclei were considered as myotubes. For statistical analysis, at least 3 populations derived from different patients were analyzed for each group.

### Acetylcholinesterase activity measurement

Acetylcholinesterase (AChE) activity measurement was performed as described before [29]. In short, the medium was carefully removed from cells grown on a 24-well plate with the immediate addition of 300 μl 0.5 mM DTNB solution (prepared in phosphate buffer, pH 7.2 with 0.1% Triton X-100). After 2 min of incubation at room temperature in the dark, 50 μl of 5.76 mM ATI (prepared in distilled water) was added. The reaction contents were incubated for 60 min at 30 °C in the dark followed by the OD measurement at 412 mM on an Anthos Zenyth 340rt microplate reader (Biochrom Ltd., Cambridge, UK). AChE activity (mUrel) was normalized per gram protein of lysed cells.

### Creatine kinase activity measurement

The medium from adherent cells grown on a 24-well plate was gently removed, and cells were washed with 1 ml of Tyrode’s salt solution (Sigma-Aldrich Co. LLC, MO, USA). Immediately afterwards, 70 μl lysis buffer was added directly onto the cells. Lysis buffer was prepared by adding 10 μl of Triton X-100 to 10 ml of dH_2_O (LC-MS-Ultrachromasol, Fluka). After 5 min incubation at 4 °C in the dark, 400 μl of CK-NAC (Thermo Scientific, MA, USA), previously dissolved by adding 10 ml dH2O, was added. The reaction was analyzed in an Anthos Zenith 340rt microplate reader (Biochrom Ltd., Cambridge, UK) set to 30 °C, by OD absorbance measurement at 340 nm. If not otherwise mentioned, OD340 nm values taken 21 min after the addition of CK-NAC were used for subsequent analysis. CK activity in mUrel was calculated according to the manufacturer’s instructions and if not otherwise mentioned normalized per gram protein of lysed cells.

### Protein quantification

Protein content of cell lysates for AChE and CK activity measurement was determined using the Pierce BCA Protein Assay Kit (Thermo Scientific, MA, USA) according to the manufacturer’s instructions by measuring the OD at 540 nm with an Anthos Zenyth 340rt microplate reader (Biochrom Ltd., Cambridge, UK).

### Standard light/fluorescence microscopy

Phase-contrast and fluorescence microscopy at × 200–600 magnification was carried out on cells cultivated on plastic cell culture vessels or glass coverslips using a Nikon Eclipse TE 2000-U microscope (Nikon Corporation, Tokyo, Japan). Representative fields were photographed with Digital Sight DS-L1 system (Nikon Corporation, Tokyo, Japan).

### Animal surgery

For both, injection of murine MPC-SMC into the pyloric sphincter as well as injection of murine MPC into the *tibialis anterior* muscle, adult female SHO-Prkdc^scid^Hr^hr^ mice were anesthetized by applying ketamine, xylazin, and acepromazin intraperitoneally. Cryopreserved murine MPC-SMC were freshly thawed, washed once with 1× PBS, and resuspended in 1× PBS to reach a final concentration of 40,000 000 cells/ml. Twenty-five microliters of the cell suspension (containing 1,000,000 cells) was then mixed with 5 μl FluoSpheres® polystyrene, 15-μm yellow-green beads (Life Technologies, Thermo Scientific, MA, USA). For MPC-SMC injection into the pyloric sphincter, a median laparotomy was performed followed by localization of the pyloric sphincter region and application of 30 μl cell-fluosphere mixture using a 30-G needle attached to a 1-ml syringe. The peritoneum muscle and skin layers were closed separately by running sutures with 6-0 Ethicon PDS plus absorbable monofilaments (Johnson & Jonson, NJ, USA). For MPC injection into the *tibialis anterior* muscle, anesthetized mice were injected with 30 μl cell-fluosphere mixture using a 28-G needle attached to a 1-ml syringe through the skin into the skeletal muscle.

### Pyloric sphincter imaging

Imaging of fresh isolated pyloric sphincter regions 12 weeks after MPC-SMC injection was performed with an IVIS Spectrum (PerkinElmer, MA, USA) using the Living Image® software version 4.5.2 (PerkinElmer, MA, USA) according to the manufacturer’s instructions. In short, pyloric sphincters of injected and control SHO mice were placed on a glass petri dish and placed within the IVIS system. Fluorescence pictures at a height of 2 cm with automated exposure times for the corresponding absorption and emission wavelengths of TdTomato and yellow Fluosphere beads were taken. Post hoc, signal intensities were adjusted in order to get rid of background signals by comparing with sphincter explants from control mice.

### Histology

For histological analysis, animals were deeply anesthetized with isoflurane and sacrificed by cervical dislocation. The tissue of interest was immediately dissected and cryo-fixed by plunging into liquid nitrogen-cooled 2-methylbutane. Tissue was cut at 15 μm on a Leica 1950 Cryostat, and slices were collected on SuperFrost plus slides and kept at − 20 °C until further processing. For immunohistological analysis, sections were fixed with 4% PFA and washed with PBS containing 0.1% Tween-20 (Sigma-Aldrich). Blocking and antibody dilution were performed using a PBS solution containing 1% bovine serum albumin fraction (Sigma-Aldrich), 0.2% fish skin gelatine (Sigma-Aldrich), and 0.1% Tween-20 (Sigma-Aldrich). Primary antibodies against tdTomato (Sicgen) or aSMA (Thermo Scientific, MA, USA) were diluted 1:100 in blocking media, and incubation was performed overnight at 4 °C. Secondary antibodies (Thermo scientific) were diluted 1:500 and applied at room temperature for 4 h. The nuclei were stained with DAPI diluted to 0.5 μg/ml working concentration (Sigma-Aldrich). Slices were subsequently mounted using Prolong Gold Antifade (Life Technologies). Fluorescence images were acquired using a LSM 710 confocal microscope and ZEN 2011 Black Software (Carl Zeiss).

### Patch-clamp analysis

Patch-clamp analysis was performed according to a previously published protocol [19], with slight experiment-specific adaptions. Procedures were conducted as follows. Electrophysiological recording was performed in a whole-cell configuration using an Axopatch 200A patch clamp amplifier (Axon Instruments, Foster City). Patch pipettes with resistances of 1 to 4 MΩ were made from borosilicate glass (GC150F-7.5, Clark Electromedical Instruments, UK) and filled with pipette solution. All data were digitized using a DIGIDATA 1200 interface (Axon Instruments, Foster City), smoothed by means of a four-pole Bessel filter, and saved to disc. Current traces were sampled at 10 kHz and filtered at 2 kHz. The pClamp software package (version 10.0 Axon Instruments, Inc.) was used for data acquisition. Microcal Origin 7.0 was used for analysis. If not otherwise mentioned, reagents were obtained from Sigma-Aldrich. Inward current of voltage-dependent Ca_v_ channels was evoked by applying 500-ms depolarizing pulses from a holding potential of − 50 to 50 mV. Superimposed current traces of K_v_ channels were evoked by step depolarizing pulses between − 80 and 60 mV in steps of 20 mV from a holding potential of − 80 mV in MPC, MPC-SMC, and hBd-SMC.

### GeneChip microarray

Total RNA of 1 × 10^6^ MPC or MSC, each cultured either in growth medium or 6 days in differentiation medium, was isolated by the RNEasy Kit (QIAGEN, Hilden, Germany) according to the manufacturers’ instructions. Sample preparation for microarray hybridization was carried out as described in the NuGEN Ovation PicoSL WTA System V2 and NUGEN Encore Biotin Module manuals (NuGEN Technologies, Inc., San Carlos, CA, USA). Hybridized arrays were washed and stained in an Affymetrix Fluidics Station FS450, and the fluorescent signals were measured with an Affymetrix GeneChip Scanner 3000 7G. The Affymetrix GeneChip Command Console v4.1.3 software controlled fluidics and scan functions. The Affymetrix Service Provider and Core Facility, “KFB - Center of Excellence for Fluorescent Bioanalytics” (Regensburg, Germany) performed the sample processing.

### Microarray data analysis

Summarized probe set signals in log2 scale were calculated by using the RMA algorithm with the Affymetrix GeneChip Expression Console v1.4 Probeset IDs with the highest log2 fold change between MPC and MPC-SMC were used for subsequent analysis and comparison to log2 fold changes between MSC and MSC-SMC. Heat maps were generated with the Multiple Expression Viewer (MeV 3.1.0) software in order to visualize log2 fold changes and perform hierarchical clustering as well as *k*-means clustering according to Euclidean distance. Genes with Log2 FC of 1 or more were considered upregulated and genes with a log2 FC of − 1 or less as downregulation.

### Quantitative data analysis

Data analysis for descriptive and inferential statistics was performed by using the GraphPad Prism software version 5.0. If not otherwise mentioned, data is presented as mean ± SD. For inferential statistics, *p* values below 0.05 were considered as statistically significant differences. *p* values below 0.05, 0.01, and 0.001 are visualized as *, ** and ***, respectively.

## Results

### MPC isolation and characterization

**Fig. 1:**
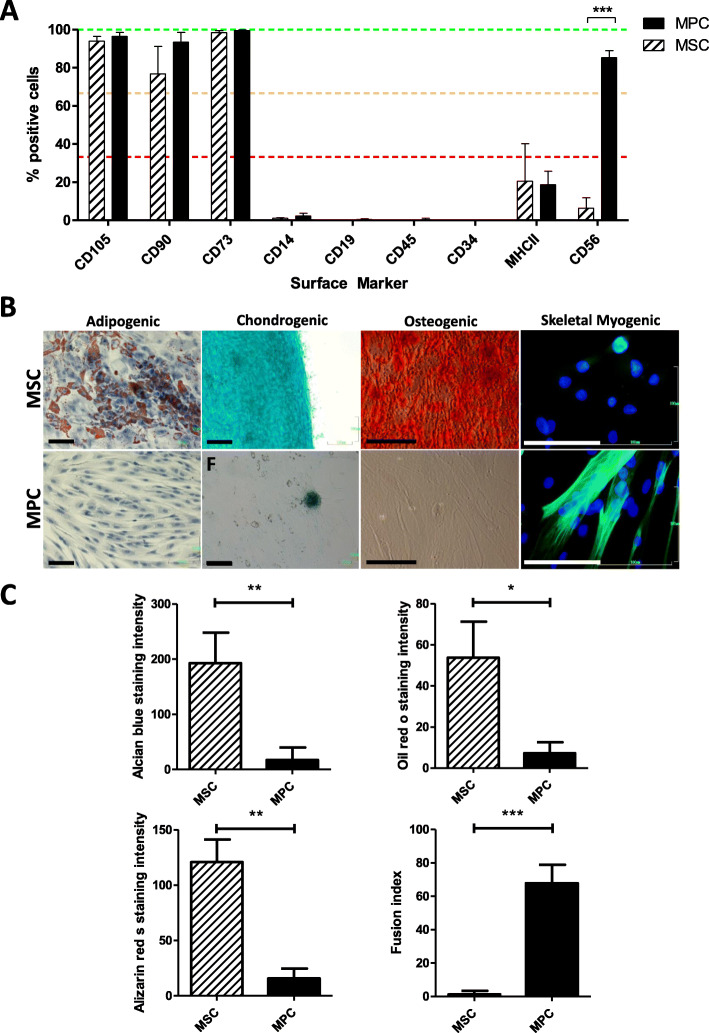
Characterization of skeletal muscle-derived MPC and MSC by cell surface marker expression and differentiation potential. **a** Surface expression of mesenchymal (CD105, CD90, CD73), myogenic (CD56), and hematopoietic (CD14, CD19, CD45, CD34, MHCII) lineage markers on both skeletal muscle-derived MPC and MSC assessed by flow cytometry. Data presented as mean ± SD of MPC and MSC from at least three individual muscle biopsies. Statistical comparison was performed by multiple unpaired *t* tests corrected for multiple testing by Holm-Sidak not assuming consistent SD (corrected *p* < 0.05 considered significant). **b** Differentiation potential of MPC and MSC assessed after in vitro differentiation to adipogenic, chondrogenic, osteogenic, and skeletal myogenic lineages by cultivation in respective differentiation media and detected by oil red o, alcian blue, alizarin red s, and anti-desmin/Hoechst staining, respectively. Representative images of at least three individual preparations are shown (scale bar = 100 μm). **c** Quantification of adipogenic, chondrogenic, osteogenic, and skeletal myogenic differentiation potential of MSC and MPC by calculation of the mean staining intensity per field of oil red o, alcian blue, or alizarin red s staining (how many fields) or by fusion index calculation of at least three individual samples, respectively

Human skeletal muscle-derived cells were isolated from adult human skeletal muscle tissue. Both CD56^+^ and CD56^−^ cells were enriched by MACS as described before [29]. CD56^+^ cells, known to be committed to the myogenic lineage [31], are referred to as myogenic progenitor cells (MPC) hereafter, while CD56^−^ cells, hypothesized to be utmost multipotent mesenchymal stromal cells, are termed MSC. We characterized MPC and MSC following isolation to confirm the efficiency of their separations (Fig. 1a). They were tested for the presence of mesenchymal lineage markers (CD105, CD90, CD73) and hematopoietic markers (CD14, CD19, CD45, CD34, MHCII), constituting the consensus minimal panel for the characterization of MSC by flow cytometry [32]. CD56^+^ MPC highly expressed all mesenchymal lineage markers tested and were negative for tested hematopoietic markers. Thus, our marker panel confirmed the mesenchymal character of CD56^+^ MPC (Fig. 1a).

Next, MPC and MSC were tested in vitro for their differentiation potential into the adipogenic, chondrogenic, osteogenic, and skeletal myogenic lineages using the appropriate culture conditions. The absence of oil red o- and alizarin red s-positive cells revealed the incapacity of MPC to differentiate into adipocytes and osteocytes, respectively (Fig. 1b, c). Furthermore, only low levels of chondrogenic differentiation occurred according to the scarce cells with alcian blue staining detected (Fig. 1b, c). In contrast, differentiation of the CD56^−^ MSC led to numerous cells positive for oil red o, alcian blue, and alizarin red s following cultivation in adipogenic, chondrogenic, and osteogenic differentiation medium, respectively (Fig. 1b, c). Quantification of staining intensities of MSC compared to MPC following differentiation in vitro revealed a significantly higher intensity in oil red o (*p* = 0.0117), alcian blue (*p* = 0.0020), and alizarin red s (*p* = 0.0012) staining. These observations confirmed the multipotent mesenchymal stromal character of CD56^−^ MSC as defined by the International Society for Cellular Therapy [32]. However, desmin-positive multinucleated myotubes were detected in MPC but not MSC cultures (Fig. 1b), and a significantly higher fusion index (*p* = 0.0007) was found within MPC compared to MSC (Fig. 1c). Hence, these results confirmed that MPC were cells of mesenchymal origin and committed to the myogenic lineage. In contrast, muscle-derived CD56^−^ MSC were capable of adipogenic, chondrogenic, and osteogenic differentiation but were not able to undergo myogenic differentiation in vitro.

### In vitro differentiation of MPC to myogenic progenitor cell-derived smooth muscle cells

**Fig. 2:**
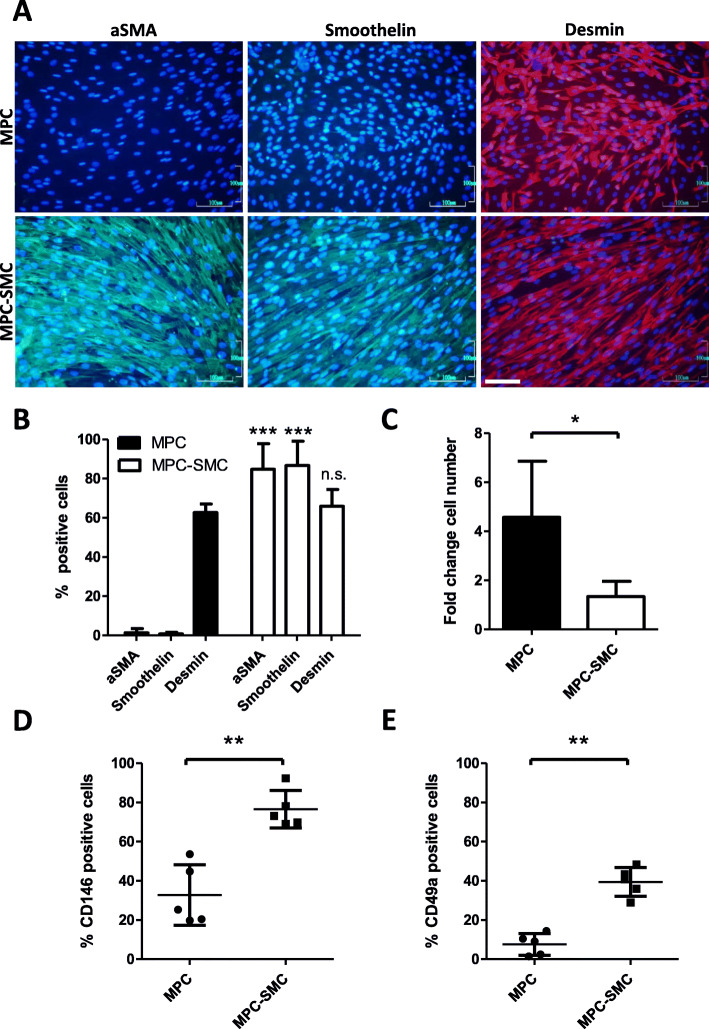
Characterization of MPC to MPC-SMC differentiation. **a** Expression of intracellular muscle-specific proteins performed by immunostainings using antibodies against aSMA, smoothelin, and desmin, each combined by nuclear staining with Hoechst dye. Representative images of cells, derived from three individual human biopsies, are shown. Scale bar (white) = 100 μm for all images. **b** Quantification of percentage MPC and MPC-SMC positive for myogenic markers aSMA, smoothelin, and desmin. **c** Fold change in cell number calculated from an initially seeded number of 500,000 cells in T175 flasks after 6 days in either growth (MPC) or smooth muscle differentiation medium (MPC-SMC). Flow cytometric quantification of **d** CD146 and **e** CD49a in populations of MPC cultivated in growth (MPC) or smooth muscle differentiation medium (MPC-SMC) for 6 days. All data (**b**–**e**) demonstrated as mean ± SD and derived from at least three individual human muscle biopsies. Statistical comparison performed between MPC and MPC-SMC by paired *t* test (**c**–**e**) or two-way ANOVA with multiple testing applying Holm-Sidak correction (**b**) considering a *p* < 0.05 as statistically significant (n.s., not significant)

In order to assess the smooth muscle differentiation potential of MPC, that were cultivated in a smooth muscle differentiation medium, which has been previously employed on multipotent mesenchymal stromal cells derived from induced pluripotent stem cells [18]. MPC-derived smooth muscle cells (MPC-SMC) were analyzed for the expression of intracellular contractile smooth muscle proteins (aSMA, smoothelin) [13, 33] as well as the general myogenic marker desmin [34] by fluorescent immunostaining. Whereas aSMA and smoothelin were hardly detectable in MPC, MPC-SMC showed detectable amounts of both proteins (Fig. 2a). The proportion of aSMA- and smoothelin-positive cells was significantly higher in MPC-SMC (84.77 ± 13.02% aSMA- and 86.70 ± 12.45% smoothelin-positive cells) compared to MPC (1.37 ± 2.11% aSMA- and 0.78 ± 0.85% smoothelin-positive cells) at a *p* value of *p* < 0.001 (aSMA) and *p* < 0.001 (smoothelin) (Fig. 2b). In contrast, no significant difference was observed in a number of desmin^+^ cells between MPC (62.68 ± 4.33%) and MPC-SMC (65.93 ± 8.58%) at a *p* value of *p* = 0.5897, suggesting smooth muscle lineage commitment of MPC-SMC without losing general myogenic fate. In addition, the proliferation of MPC was addressed following 6 days of culture in growth or smooth muscle differentiation medium. The increase in the number of cells in smooth muscle differentiation medium (MPC-SMC) was significantly lower (*p* = 0.0179) during the 6-day cultivation period compared to MPC in growth medium (Fig. 2c), which is consistent with the observed switch to differentiation towards a post-mitotic phenotype. Finally, the expression of CD146 and CD49a, associated with the vascular smooth muscle commitment of MSC [35] and expressed during smooth muscle development [36], greatly increased in MPC when differentiated to MPC-SMC by cultivation for 6 days in smooth muscle differentiation medium. Percentage of both CD146 and CD49a surface marker-positive cells were significantly higher at a *p* value of *p* = 0.0033 (CD146) and *p* = 0.0031 (CD49a) in MPC-SMC (76.53 ± 9.56% CD146- and 39.49 ± 7.40% CD49a-positive cells) than in MPC (32.78 ± 15.46% CD146- and 7.59 ± 5.51% CD49a-positive cells) (Fig. 2d, e and S1 Fig.).

**Fig. 3:**
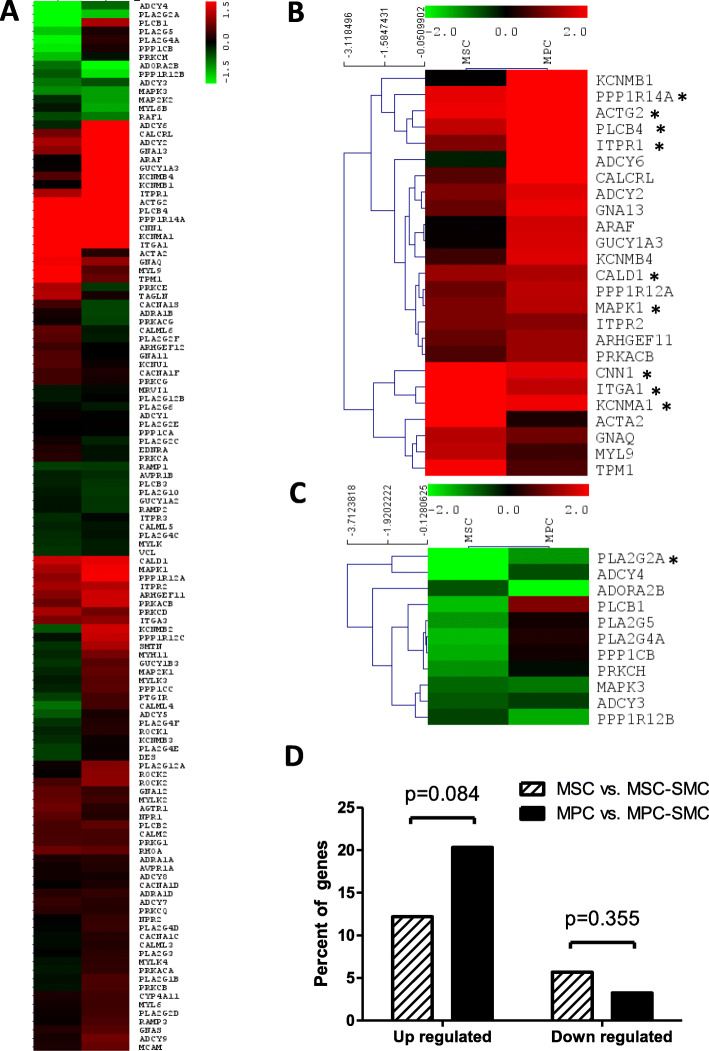
Change in the gene expression of MPC and MSC during SMC differentiation. Changes in the gene expression were assessed by microarray analysis of RNA isolated from MSC, MPC, MSC-SMC, and MPC-SMC. Cells derived from two individual human muscle biopsies were cultivated in growth (MSC and MPC) or smooth muscle differentiation medium (MSC-SMC and MPC-SMC) for 6 days, respectively. **a** Hierarchical clustering (Euclidean distance) of Log2 fold changes of smooth muscle-associated genes in MSC vs MSC-SMC (MSC) and MPC vs MPC-SMC (MPC). The cluster of genes similarly **b** upregulated or **c** downregulated in both MSC and MPC upon SMC differentiation obtained by *k*-means clustering. *Genes which in both cell types were either log2 FC ≥ 1 or log2 FC ≤ − 1 up- or downregulated. **d** Percent of tested genes calculated to be upregulated or downregulated during MSC to MSC-SMC or MPC to MPC-SMC differentiation. Statistical comparison was performed by chi-squared test considering a *p* value below 0.05 as significant

A microarray analysis of differentiating MPC was carried out to monitor the changes in gene expression associated with SMC differentiation [35, 37, 38], myogenic commitment [12], and smooth muscle contraction [39]. Changes in the gene expression occurring when MPC were differentiated to MPC-SMC were compared to those when MSC were differentiated to MSC-SMC, both by cultivation in smooth muscle differentiation medium. We observed that 25 genes (20.33%) of the 123 tested genes were up- and 4 genes (3.25%) downregulated during MPC to MPC-SMC differentiation. Among the genes upregulated in MPC-SMC, we confirmed our observation of an increase in CD49a (ITGA1) and smoothelin (SMTN) expression. Further, genes upregulated belong to the KEGG cluster and other known smooth muscle marker genes: PPP1R14A, KCNMB1, PLCB4, ACTG2, ITPR1, ADCY6, CALCRL, KCNMA1, GNA13, CNN1, ADCY2, KCNMB4, GUCY1A3, ARAF, PPP1R12A, MAPK1, CALD1, KCNMB2, PRKACB, ARHGEF11, PPP1R12C, ITPR2, and PLCB1 (Fig. 3a). Although CD146 (MCAM) surface protein expression was found to be upregulated in MPC-SMC, MCAM gene expression was not upregulated in the microarray experiments, suggesting a post-transcriptional regulation. Further, analysis of log2 FC changed gene expressions in MPC and MSC during differentiation to MPC-SMC and MSC-SMC led to the identification of up- and downregulated genes that were shared with differentiating MPC and MSC (Fig. 3b, c). Whereas PP1R14A, ACTG2, PLCB4, ITPR1, MAPK1, CNN1, ITGA1, and KCNMA1 were upregulated in MPC and MSC, PLA2G2A was downregulated in both cell types. Overall, 75.61% of genes analyzed were regulated similarly (i.e., up-, down-, stably regulated) in MPC and MSC upon differentiation using smooth muscle differentiation medium. More upregulated genes and less downregulated genes were found in differentiating MPC (20.33% up- and 3.25% downregulated) than differentiating MSC (12.20% up- and 5.69% downregulated). However, no significant difference in percent up- or downregulated genes was found between MPC and MSC. Taken together, these results suggest a differentiation efficiency of MPC to MPC-SMC being at least as good as MSC to MSC-SMC differentiation efficiency.

The smooth muscle phenotype of MPC-SMC and MSC-SMC was further compared to human bladder smooth muscle cells (hBd-SMC), following cultivation in smooth muscle differentiation medium for 6 days, by immunostaining. Immunodetection was performed against the smooth muscle marker proteins aSMA and smooth muscle myosin heavy chain (SM-MHC), as well as for desmin, a general myogenic marker, and vimentin, a contraction associated protein [40]. MPC-SMC, MSC-SMC, and hBd-SMC expressed aSMA, SM-MHC, and vimentin. Additionally, MPC-SMC and hBd-SMC expressed desmin, which was not found in MSC-SMC (S2 Fig.). This observation substantiates that MPC-SMC differentiate to a smooth muscle phenotype resembling the one of hBd-SMC.

### Stability of the MPC-SMC phenotype

**Fig. 4:**
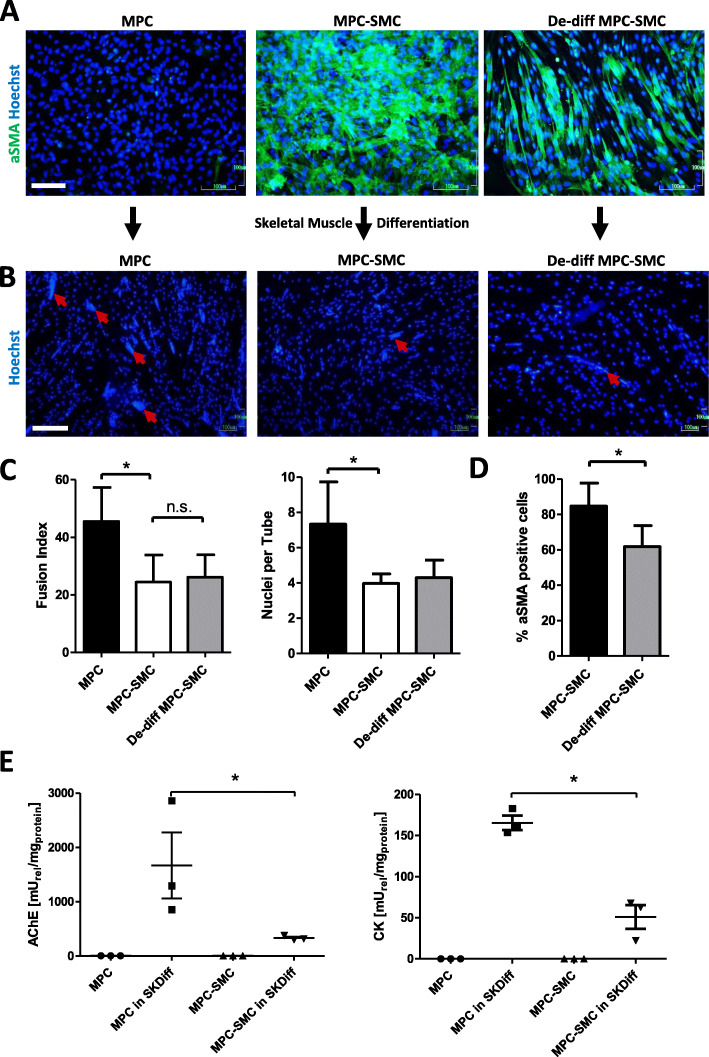
Stability of smooth muscle differentiation program of MPC-SMC. **a** Smooth muscle lineage marker (aSMA) expression stained by fluorescent immunostaining was compared between MPC, MPC-SMC, and MPC-SMC de-differentiated by cultivation for 3 days in growth medium following cultivation in smooth muscle differentiation medium (De-diff MPC-SMC). Representative images of MPC, MPC-SMC, and De-diff MPC-SMC derived from at least three different skeletal muscle biopsies are shown. Scale bar = 100 μm. **b** Formation of myotubes (red arrows) observed by fluorescence microscopy following Hoechst33342 staining for visualization of the nuclei as well as **c** quantification of fusion index (FI) and the number of nuclei was compared between MPC, MPC-SMC, and De-diff MPC-SMC each cultivated in skeletal muscle differentiation medium for 6 days. Scale bar = 200 μm. **d** Quantification of percent aSMA-positive cells in MPC-SMC and De-diff MPC-SMC was performed. **e** Comparison of AChE and CK enzyme activity between MPC, MPC-SMC, and MPC in skeletal muscle differentiation medium (MPC in SKDiff) and MPC-SMC in skeletal muscle differentiation medium (MPC-SMC in SKDiff). All data presented as mean ± SD of cells derived from at least three individual human muscle biopsies. Statistical analysis performed by paired *t* test considering *p* < 0.05 as significant

To assess whether the phenotype of MPC-SMC is irreversible, aSMA protein expression was evaluated in MPC maintained in the growth medium, MPC cultivated in smooth muscle differentiation medium for 6 days (MPC-SMC) and in MPC-SMC that were cultured 3 additional days back in growth medium (De-diff MPC-SMC). As shown in Fig. 4a, MPC-SMC and De-diff MPC-SMC prominently expressed aSMA, in contrast to MPC. Significantly fewer aSMA-positive cells were found however in De-diff MPC-SMC (61.87 ± 11.84) compared to MPC-SMC (81.92% ± 16.50, *p* = 0.0196), thus demonstrating either a partial reversion of the smooth muscle phenotype or proliferation of MPC that did not differentiate to MPC-SMC in the first place and remained aSMA negative (Fig. 4d). Nevertheless, a substantial amount (> 60%) of the cells maintain their smooth muscle lineage commitment.

In order to address the skeletal muscle differentiation potential of MPC-SMC, as compared to MPC and De-diff MPC-SMC, their fusion competence based on the formation of multinucleated myotubes was assessed. Following the cultivation of all three cell types in skeletal muscle differentiation medium for 6 days, calculation of fusion index (FI) and quantification of the number of nuclei per tube were performed. Our results showed that MPC formed numerous multinucleated myotubes, whereas MPC-SMC and De-diff MPC-SMC formed only few myotubes (Fig. 4b, red arrows). Quantification of the FI demonstrated that significantly more MPC underwent skeletal myogenesis compared to MPC-SMC, revealing a decrease in the skeletal myogenic potential of MPC upon differentiation to MPC-SMC (Fig. 4c). Further, tubes formed by MPC-SMC contained significantly fewer nuclei than tubes formed by MPC (Fig. 4c). Additionally, no significant difference in terms of FI was detected between MPC-SMC and De-diff MPC-SMC (Fig. 4c). Moreover, acetylcholinesterase (AChE) and creatine kinase (CK), two enzymes upregulated during skeletal myogenesis [41, 42], were analyzed. Low AChE and CK activities were detectable in MPC and MPC-SMC. Upon induction of skeletal muscle differentiation, however, a significantly lower AChE and CK activity were found in MPC-SMC compared to MPC, suggesting a lower skeletal muscle differentiation potential of MPC-SMC (Fig. 4e).

### Functional analysis of MPC-derived SMC in vitro

**Fig. 5:**
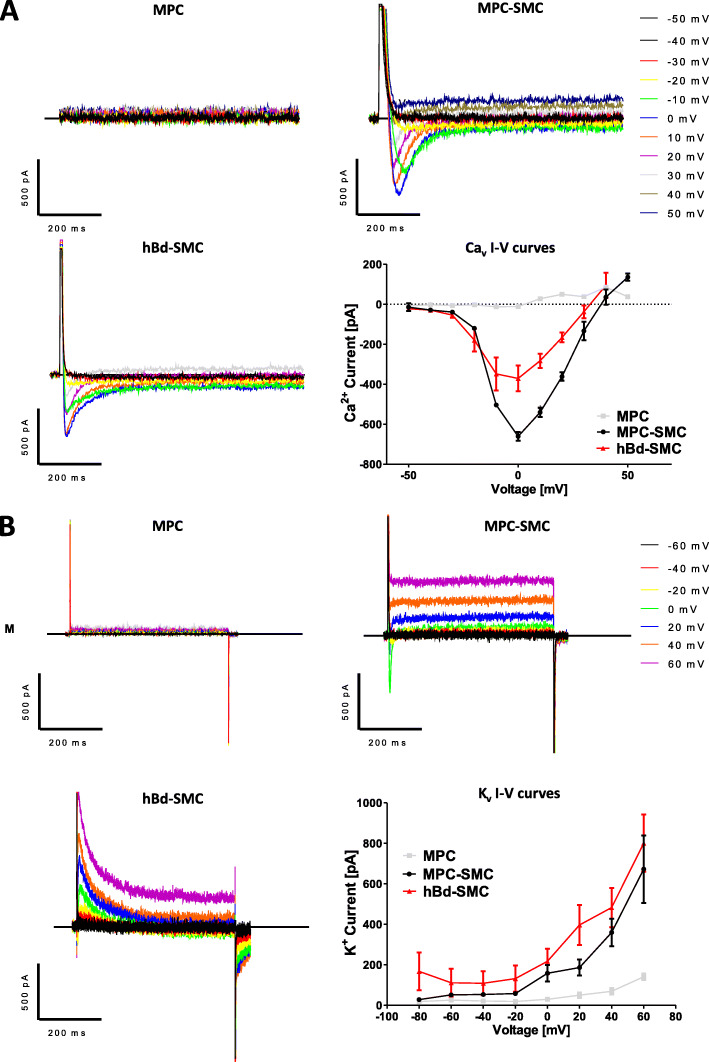
Expression of functional ion channels during MPC to SMC differentiation. Analysis of voltage-dependent **a** inward calcium and **b** outward potassium currents in MPC, MPC-SMC, and hBd-SMC. Representative inward and outward voltage-dependent traces of MPC, MPC-SMC, and hBd-SMC are shown. Impedance-voltage (I-V) curves of each at least three cells of MPC, MPC-SMC, and hBd-SMC are demonstrating presence of functional **a** Ca_v_ and **b** K_v_ channels in MPC-SMC but not MPC

Since MPC were showing profound changes in gene and protein expression upon smooth muscle differentiation, we hypothesized that smooth muscle differentiation of MPC in vitro will also lead to functional maturation. It has been shown that TGFb1-induced differentiation of adipose tissue-derived multipotent mesenchymal stromal cells leads to the expression of functional voltage-sensitive calcium and potassium channels [19]. Therefore, the presence of functional voltage-sensitive Ca_v_ and K_v_ channels was explored by patch-clamp analysis on MPC maintained in growth medium or differentiated to MPC-SMC by cultivation in smooth muscle differentiation medium for 6 days. As a control, hBd-SMC cultivated in smooth muscle differentiation medium for 6 days were analyzed in terms of their functional voltage-sensitive channels by patch-clamp analysis as well. MPC lacked both inward and outward currents from voltage-dependent calcium and voltage-dependent potassium channels. In contrast, MPC-SMC and hBd-SMC showed voltage-sensitive inward and outward currents for both cations (Fig. 5a, b).

Park et al. showed [19] that together with the expression of functional ion channels, in vitro contractility increased during smooth muscle differentiation of adipose-derived MSC. Therefore, both MPC and MPC-SMC were tested for contractility in a collagen gel lattice contraction assays. We also included in this analysis MSC, MSC-SMC, and hBd-SMCs. When MPC were differentiated to MPC-SMC, contractility increased from 22.11 ± 12.77% (MPC) to 46.57 ± 6.75% (MPC-SMC) (*p* = 0.0118). MSC-SMC were found to contract similarly (53.54 ± 22.53% gel contraction) compared to MPC-SMC (adjusted *p* = 0.5534). hBd-SMC were found to exhibit the highest contractility (85.84 ± 2.57%) and significantly more than MPC-SMC (*p* value adjusted for multiple testing, *p* = 0.0006) (S3 Fig.).

These results together suggest that upon smooth muscle differentiation, MPC functionally maturate to MPC-SMC in vitro.

### Engraftment of MPC-derived SMC into smooth muscle tissue in vivo

**Fig. 6:**
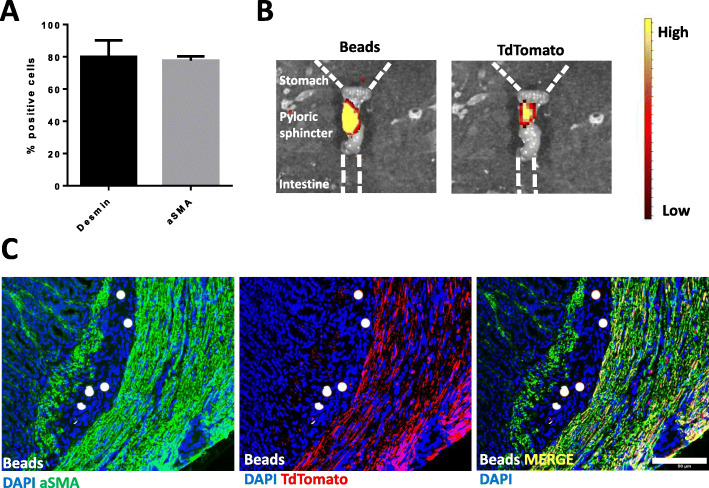
Engraftment of mMPC-derived SMC into smooth muscle tissue in vivo. **a** Quantification of percent desmin- and aSMA-positive cells performed by immunofluorescence staining of two preparations of mMPC-SMC. **b** Fluorescence signal detection of co-injected fluorescent beads and TdTomato transgene expressing cells localized in the intact pyloric sphincter muscle by IVIS imaging ex situ. **c** Tissue engraftment and aSMA protein expression of TdTomato-positive mMPC-SMC in pyloric sphincter histological sections of intramuscular-injected SMC 12 weeks after implantation. Representative images of *n* = 8 injected mice

We next addressed whether MPC-SMC are able to engraft into smooth muscle tissue in vivo. To this end, we isolated murine myogenic progenitor cells (mMPC) from transgenic TdTomato reporter mice and differentiated those mMPC to mMPC-SMC in vitro as described above. mMPC were found to be desmin+ and capable of fusing to multinucleated myotubes exhibiting AChE and CK activity following skeletal muscle differentiation (S4 Fig. A-D). In addition, mMPC were able to fuse to and/or with host myofibers following implantation into the skeletal muscle tissue of immunodeficient mice (S4 Fig. E, F). Following smooth muscle differentiation of mMPC, resulting mMPC-SMC were highly positive for desmin (77.60 ± 2.69%) and aSMA (80.00 ± 14.48%) (Fig. 6a), similar to human MPC-SMC, described above. mMPC-SMC, together with fluorescent beads, were transplanted into the pyloric sphincter of immunodeficient SHO mice. mMPC-SMC expressing the reporter TdTomato were detectable at the site of injection 12 weeks post-implantation, suggesting the engraftment of mMPC-SMC in the pyloric sphincter (Fig. 6b). Immunohistological examination for TdTomato and aSMA proteins revealed that TdTomato expressing cells were found within the pyloric sphincter circular muscle as well as *muscularis mucosa* (Fig. 6c). In addition, TdTomato-positive cells that are located within the smooth muscle layer of the pyloric sphincter expressed aSMA demonstrate the smooth muscle phenotype of mMPC-SMC after engraftment (Fig. 6c).

## Discussion

The present study provides to our knowledge the first evidence that skeletal muscle-derived MPC can be differentiated into smooth muscle cells in vitro and thereby express contractile proteins, surface markers, as well as functional voltage-gated ion channels associated with smooth muscle lineage commitment. Furthermore, we report that this differentiation is stable even when MPC-SMC are removed from smooth muscle differentiating conditions. Importantly, we underscore the potential of MPC-SMC for sphincter regeneration by demonstrating the engraftment, survival, and contractile protein expression of MPC-SMC following transplantation into the murine pyloric sphincter in vivo.

To date, different sources of human cells capable of in vitro differentiation towards the smooth muscle lineage have been described, whereby most studies included induced pluripotent stem cells (iPSC) and MSC [18, 19, 35]. Both iPSC and MSC can be isolated without ethical concerns and harbor great differentiation potential; high proliferative capacities are easily accessible as well as clinically suitable for autologous treatment approaches [43, 44]. iPSC especially are promising in their smooth muscle differentiation potential and functionality in vitro [18], and iPSC-derived smooth muscle progenitor cells demonstrated urethral sphincter regenerative potential in vivo [45]. However, concerns on safety, such as genetic instability and teratoma formation, still dampen their way into the clinics [43]. Adult MSC-derived cell products did not cause major health concerns in the majority of clinical trials [46]. They have been shown effective for anal sphincter muscle regeneration in animal models [47], although their clinical efficacy remains elusive and the mode of action is yet to be elucidated. Primary smooth muscle cells were successfully used for smooth muscle regeneration in a passive fecal incontinence animal model [48]. However, these cells are hardly accessible for autologous treatment in humans, and moreover, they are heterogeneous in nature and may have limited in proliferative capacity [49]. Therefore, primary smooth muscle cells do not qualify as a promising cell therapeutic candidate for smooth muscle regeneration in humans. In contrast, skeletal muscle-derived cells are highly proliferative [50], and CD56^+^ cells have been shown to be safe and effective in clinical trials to treat external anal sphincter-associated (skeletal muscle) fecal incontinence [21, 22, 24]. With this study, we demonstrate that CD56^+^ skeletal muscle-derived cells (MPC) also possess the potential to differentiate into functional smooth muscle in vitro, being able to engraft into the pyloric sphincter smooth muscle, following intramuscular implantation. Thus, skeletal muscle-derived CD56^+^ MPC have both skeletal and smooth muscle regenerative potential and therefore constitute a unique and powerful cell source for cell therapy in patients with defects in both skeletal and/or smooth muscle sphincters.

A previous study reported the capacity of CD34^+^/CD56^+^ muscle-derived cells having smooth muscle differentiation potential in vitro [28]. CD34 expression was described in muscle-derived stem cells and quiescent satellite cells [51], but its expression becomes heterogeneous during activation and proliferation [52]. Thus, it became questionable if CD34^−^ muscle-derived cells maintain their smooth muscle differentiation potential. MPC isolated in the present study were found to be CD34^−^, and thus, our results for the first time demonstrate that CD34^−^/CD56^+^ MPC maintain smooth muscle differentiation potential. Our results showed for the first time to our knowledge that treatment of CD34^−^ /CD56^+^ MPC in smooth muscle differentiation medium containing TGFb1 and heparin induced expression of smooth muscle-associated proteins (aSMA and smoothelin) and maintained expression of the myogenic marker desmin. Convincingly, CD146, a marker associated with the vascular lineage commitment of MSC [35] and also detected in synthetic human bladder-derived smooth muscle cells (s-hBd-SMC), and CD49a (ITGA1), a marker dramatically increased in smooth muscle containing tissue during development [36] and detected in contractile human bladder-derived smooth muscle cells, were found in MPC-SMC. This suggests that MPC-SMC adopt surface marker profile of smooth muscle cells. Furthermore, MPC to MPC-SMC differentiation was shown herein to reduce cell proliferation consistent with differentiation and findings of others that CD146^+^ smooth muscle lineage-committed cells proliferate significantly slower than CD146^−^ cells [35].

In our study, we demonstrated that MPC-derived MPC-SMC also express functional calcium and potassium channels. Furthermore, a comparison of changes in the pattern of gene expression between MPC and MSC during smooth muscle differentiation revealed highly similar differentiation efficiency. This suggests MPC are at least as good as MSC to generate smooth muscle cells in vitro. Although an upregulated protein expression of aSMA could be detected in MPC-SMC, we detected upregulation of ACTA2 gene expression encoding aSMA only following the smooth muscle cell differentiation of MSC, but not MPC. The higher amount of aSMA protein-positive cells MPC-SMC compared to MPC could result from (i) an increased polymerization of globular actin (G-aSMA) into filamentous actin (F-aSMA), of which only the latter is detectable by aSMA antibodies [53], or (ii) post-transcriptional regulation of aSMA protein expression during MPC to MPC-SMC differentiation.

Contractility of smooth muscle cells is a prerequisite for their functionality in vivo, and an increase in contractility of in vitro generated smooth muscle cells was already confirmed for adipose- and bone marrow MSC-derived smooth muscle cells [19, 35]. Our results go along with these findings as skeletal muscle-derived MSC found herein increased in contractility upon in vitro smooth muscle differentiation to MSC-SMC. Strikingly, we could demonstrate for the first time increased contractility of MPC following in vitro differentiation to MPC-SMC, comparable to the contractility of MSC-SMC. However, the contractility of SMC was not yet as pronounced as of hBd-SMC, suggesting that MPC-SMC are on their way to become fully mature contractile smooth muscle cells.

Previous studies used muscle-derived cells for the treatment of detrusor muscle dysfunction [27, 54]. Although improvement of bladder function following cryoinjury was reported, injected cells also formed skeletal muscle fibers within the smooth muscle tissue [27]. In this case, improvement of bladder function could be explained by increasing the total amount of tissue and thus compensate for lost tissue. However, this mere replacement of tissue by cells not having the same functionality as lost cells might not be regenerative. In contrast, we provide evidence that murine MPC-SMC generated according to our protocol, survived within the pyloric sphincter of immunodeficient mice, and express aSMA. Importantly, we did not find skeletal myofibers within the smooth muscle tissue of the pyloric sphincter following transplantation, suggesting smooth muscle lineage commitment stability. Moreover, our in vitro data demonstrated that human MPC-SMC exhibit less skeletal myogenic differentiation potential (fusion competence, AChE, and CK activity) than MPC, which might put them in favor as a therapy for smooth muscle regeneration.

The smooth muscle differentiation potential of MPC in vitro as well as the integration capacity of MPC-SMC in the sphincter muscle makes them an auspicious candidate for regenerative therapy in conditions such as passive fecal incontinence. Further studies addressing the safety and efficacy of MPC-SMC in smooth muscle sphincter regeneration will be required. The establishment of faithful small and large animal disease models for sphincter regeneration will be pivotal for gathering new insight into the functional regenerative potentials of MPC-SMC and the establishment of effective smooth muscle regeneration therapies.

## Conclusions

Human skeletal muscle-derived myogenic progenitor cells demonstrate smooth muscle differentiation potential in vitro. Smooth muscle differentiation of myogenic progenitor cells was induced by cultivating them in TGFb1 and heparin-containing medium. Resulting smooth muscle cells demonstrate increased expression of smooth muscle marker genes and proteins, as well as functional voltage-gated cation channels and in vitro contractility. These in vitro-generated smooth muscle cells engraft in sphincter smooth muscle tissue following intramuscular implantation in vivo*.* Taken together, myogenic progenitor cell-derived smooth muscle cells could be a promising medicinal product for the treatment of smooth muscle sphincter-related disorders such as passive fecal incontinence.

## Supplementary information

**Additional file 1: S1 Fig**. Surface marker expression in synthetic and contractile hBd-SMC compared to MPC and MPC-SMC. Immunocytochemistry against CD146 or CD49a combined each with hematoxylin staining on MPC and SMCs cultivated in either growth medium (MPC and s-hBd-SMC) or smooth muscle differentiation medium (MPC-SMC and hBd-SMC) for 6 days. Representative images of MPC/MPC-SMC from at least two individual muscle biopsies and of two individual experiments (hBd-SMC and s-hBd-SMC). Scale bar = 100 μm.

**Additional file 2: S2 Fig**. Contractile protein expression in bladder smooth muscle cells compared to MSC-SMC and MPC-SMC. Human skeletal muscle-derived MPC-SMC and MSC-SMC were compared to bladder smooth muscle-derive hBd-SMC by immunostaining for aSMA (green), SM-MHC (green), desmin (red) and vimentin (green), each combined by nuclear staining with Hoechst dye (blue). Representative images from at least three individual experiments are shown. Scale bars = 100 μm.

**Additional file 3: S3 Fig.** Contractility measurements in collagen gel lattices. Contractility of MPC, MSC, MPC-SMC and MSC-SMC as well as hBd-SMC was quantified by collagen gel lattice contraction. (A) Percent gel contraction from original size within 48 h of cells is shown in bar graphs. Data presented as mean ± SEM of cell preparations from each at least three individual human muscle biopsies (MSC, MPC) or three individual experiments (hBd-SMC). (B) Representative stereomicroscopic images of the collagen gels with embedded MSC, MSC and SMC each derived thereof as well as hBd-SMC in wells of a 24-well plate 48 h after gel formation.

**Additional file 4: S4 Fig.** Characterization of murine MPC and MPC-SMC. (A) AChE and (B) CK activity was measured by enzyme kinetics and is represented by OD412nm and OD340nm at specific time points (AChE: 60 min, CK: 10 min). Enzyme activities were compared between skeletal muscle-derived mMPC and non-myogenic cells after 6 days in skeletal muscle differentiation medium. (C) Desmin expression of mMPC visualized by immunofluorescence staining. (D) Formation of multinucleated myotubes by mMPC during differentiation in skeletal muscle differentiation medium for six days in vitro was observed. TdTomato and nuclei were stained on histological cross sections of *tibialis anterior* muscles of (E) control untreated mice and (F) SHO mice 70 days after intramuscular injection with TdTomato mMPC and fluorescent beads. Scale bar = 100 μm.

**Additional file 5:** Supporting methods.

## Data Availability

All data generated or analyzed during this study are included in this published article and its supplementary information files.

## Abbreviations

AChE: Acetylcholinesterase
aSMA: Alpha smooth muscle actin
ATI: Acetylthiocholine
Ca_v_: Voltage-gated calcium channels
CD: Cluster of differentiation
CK: Creatine kinase
DTNB: 5,5′-Dithiobis-(2-nitrobenzoic acid)
F-aSMA: Filamentous alpha smooth muscle actin
FC: Fold change
FI: Fusion index
FITC: Fluorescein isothiocyanate
G-aSMA: Globular alpha smooth muscle actin
GMP: Good Manufacturing Practice
hBd-SMC: Contractile human bladder-derived smooth muscle cells
HBd-SMC-c: Human bladder-derived smooth muscle cells
iPSC: Induced pluripotent stem cells
K_v_: Voltage-gated potassium channels
MACS: Magnetic-activated cell sorting
mMPC: Murine myogenic progenitor cells
MPC: Myogenic progenitor cells
MPC-SMC: Myogenic progenitor cell-derived smooth muscle cells
MSC: Multipotent mesenchymal stromal cells
MSC-SMC: Multipotent mesenchymal stromal cell-derived smooth muscle cell
n.s.: Not significant
PE: Phycoerythrin
SEM: Standard error of the mean
SD: Standard deviation
s-hBd-SMC: Synthetic human bladder-derived smooth muscle cells
SMC: Smooth muscle cells
SM-MHC: Smooth muscle myosin heavy chain
SMTN: Smoothelin
TGFb: Transforming growth factor beta
