# Supplementary figures and images for: Generation of myogenic progenitor cell-derived smooth muscle cells for sphincter regeneration
Source: Stem Cell Res Ther. 2020 Jun 12;11:233. doi: 10.1186/s13287-020-01749-w (PMC7291744; doi:10.1186/s13287-020-01749-w)

CD146

CD49a

MPC

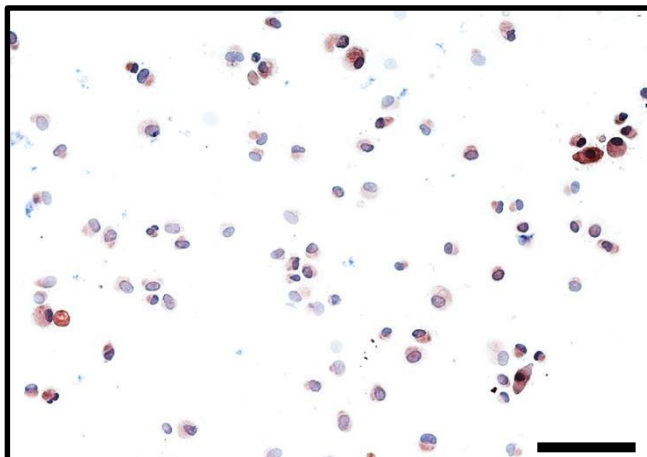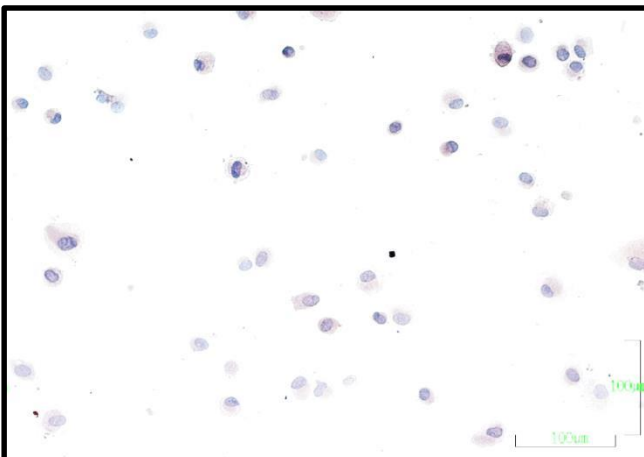

MPC-SMC

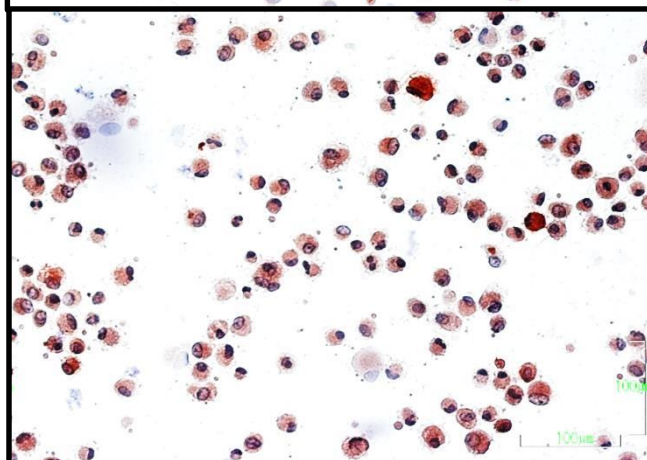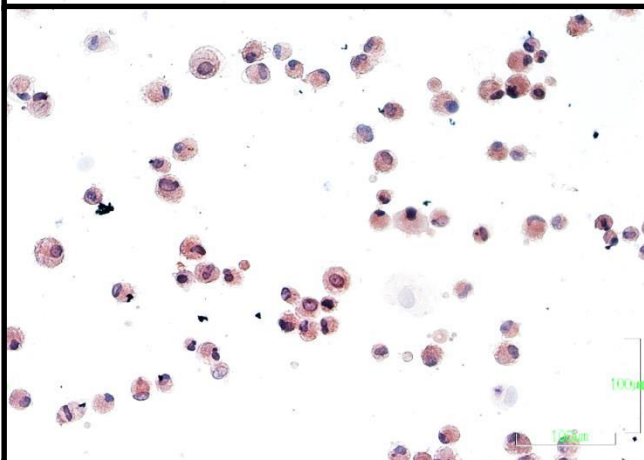

s-hBd-SMC

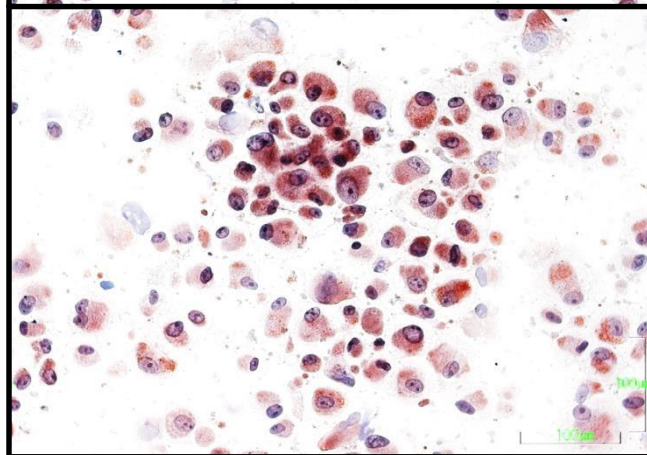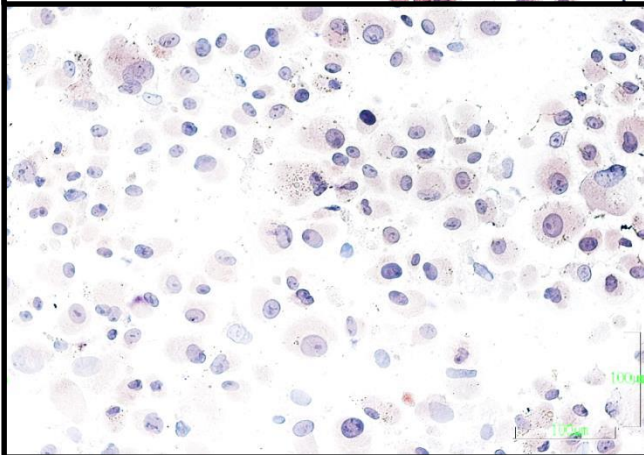

hBd-SMC

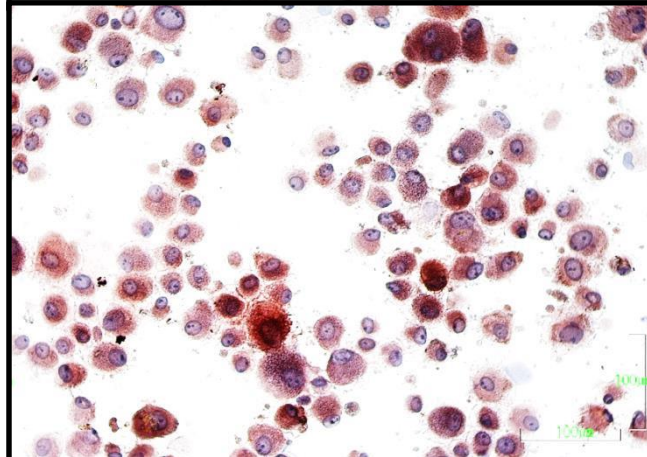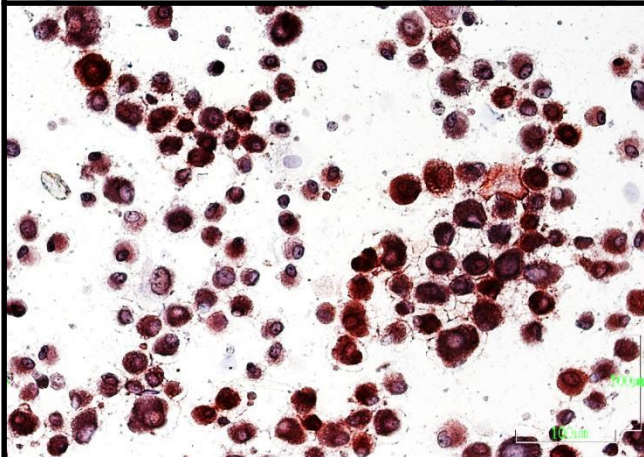

Supplement: Supplementary file 1 — Additional file 1: S1 Fig. Surface marker expression in synthetic and contractile hBd-SMC compared to MPC and MPC-SMC. Immunocytochemistry against CD146 or CD49a combined each with hematoxylin staining on MPC and SMCs cultivated in either growth medium (MPC and s-hBd-SMC) or smooth muscle differentiation medium (MPC-SMC and hBd-SMC) for 6 days. Representative images of MPC/MPC-SMC from at least two individual muscle biopsies and of two individual experiments (hBd-SMC and s-hBd-SMC). Scale bar = 100 μm. [file 13287_2020_1749_MOESM1_ESM.pdf]

aSMA

SM-MHC

Desmin

Vimentin

MPC-iSMC

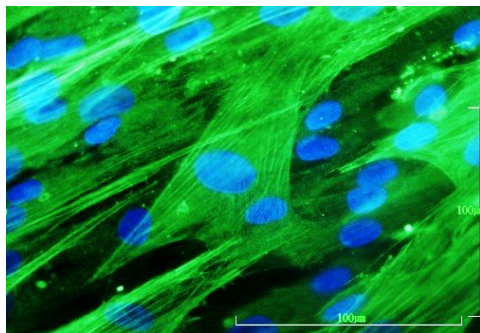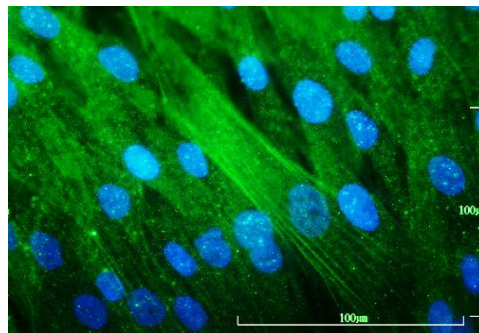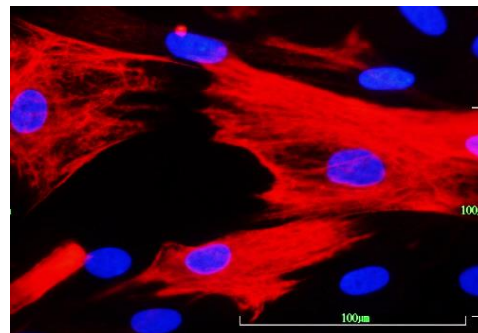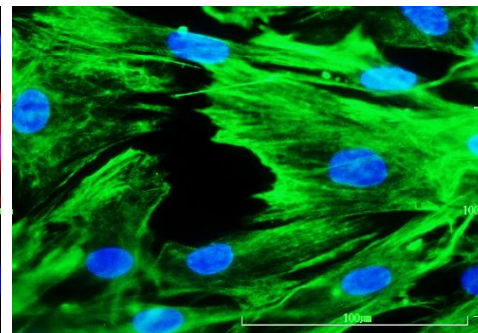

MSC-SMC

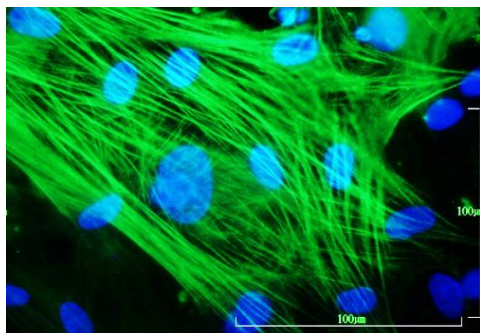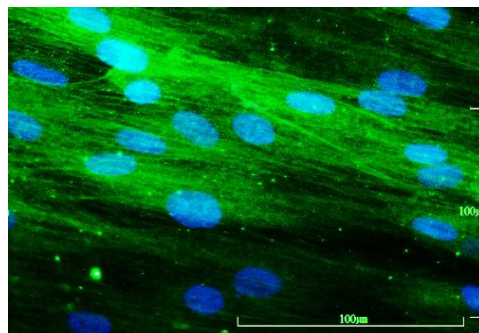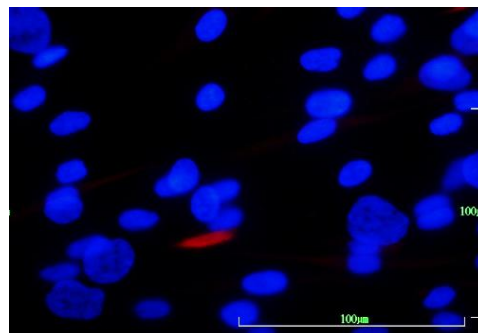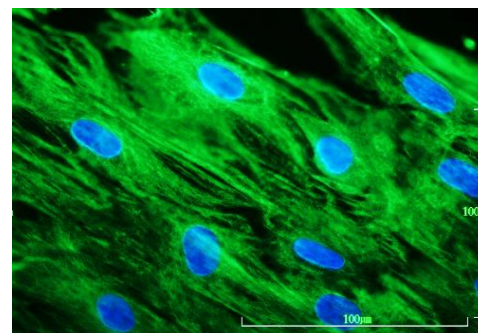

hBd-SMC

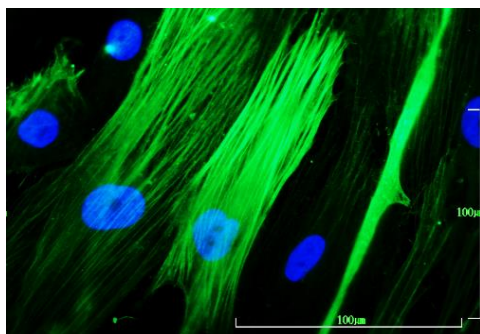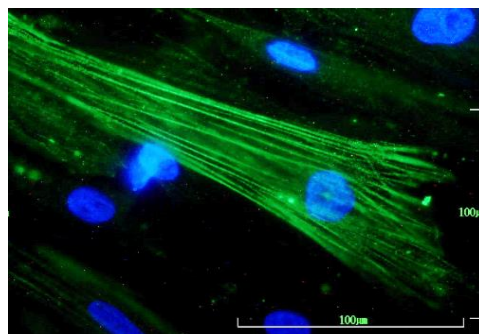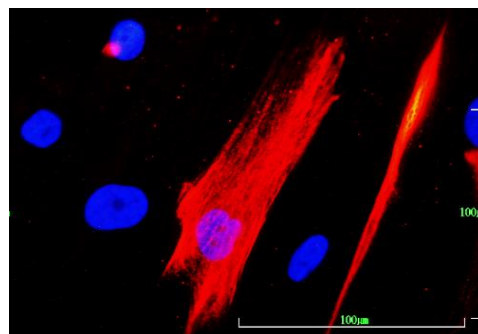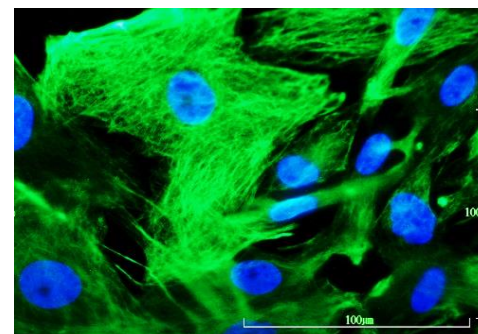

Supplement: Supplementary file 2 — Additional file 2: S2 Fig. Contractile protein expression in bladder smooth muscle cells compared to MSC-SMC and MPC-SMC. Human skeletal muscle-derived MPC-SMC and MSC-SMC were compared to bladder smooth muscle-derive hBd-SMC by immunostaining for aSMA (green), SM-MHC (green), desmin (red) and vimentin (green), each combined by nuclear staining with Hoechst dye (blue). Representative images from at least three individual experiments are shown. Scale bars = 100 μm. [file 13287_2020_1749_MOESM2_ESM.pdf]

**A**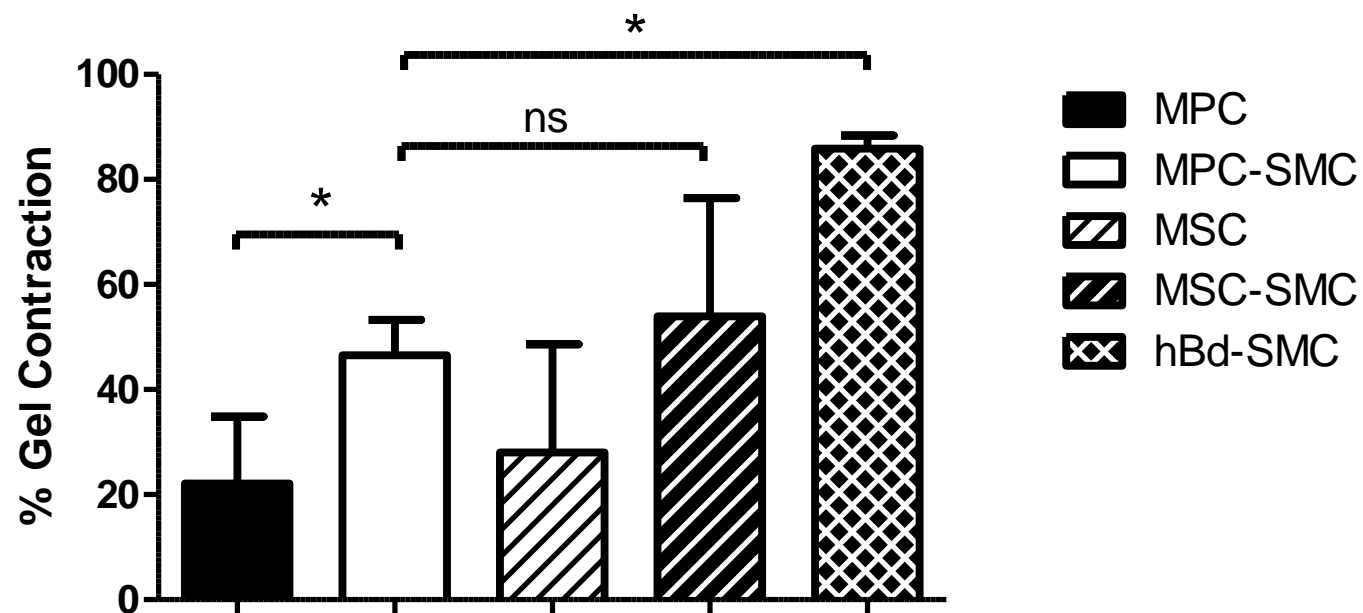**B**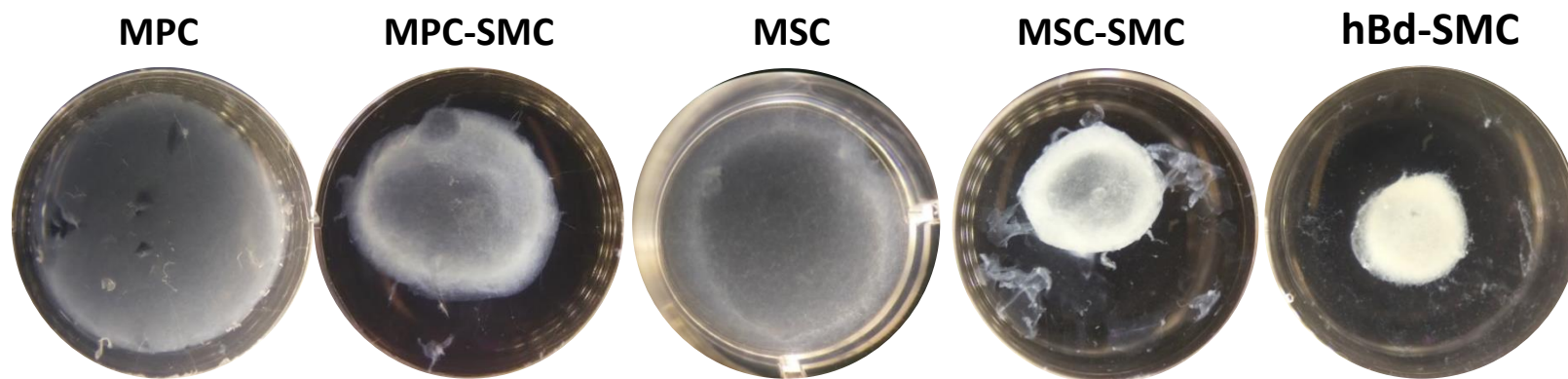

Supplement: Supplementary file 3 — Additional file 3: S3 Fig. Contractility measurements in collagen gel lattices. Contractility of MPC, MSC, MPC-SMC and MSC-SMC as well as hBd-SMC was quantified by collagen gel lattice contraction. (A) Percent gel contraction from original size within 48 h of cells is shown in bar graphs. Data presented as mean ± SEM of cell preparations from each at least three individual human muscle biopsies (MSC, MPC) or three individual experiments (hBd-SMC). (B) Representative stereomicroscopic images of the collagen gels with embedded MSC, MSC and SMC each derived thereof as well as hBd-SMC in wells of a 24-well plate 48 h after gel formation. [file 13287_2020_1749_MOESM3_ESM.pdf]

**A**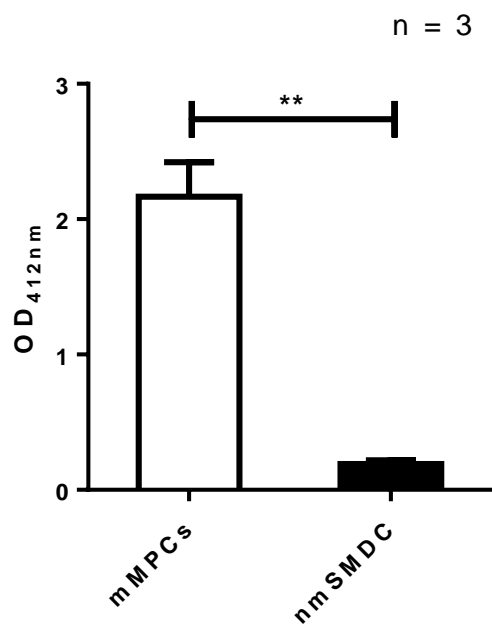**B**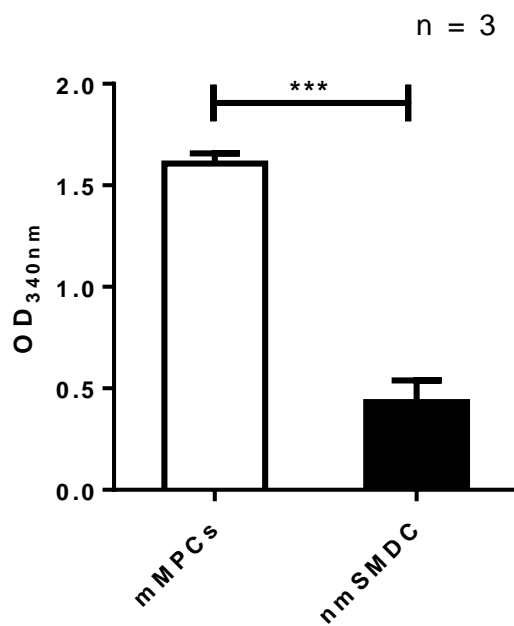**C**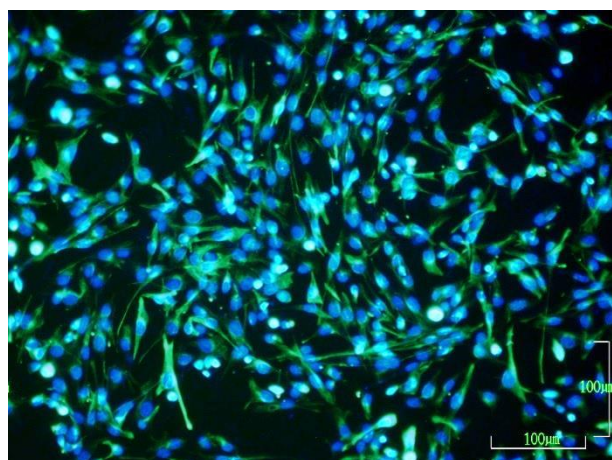

Desmin Hoechst

**D**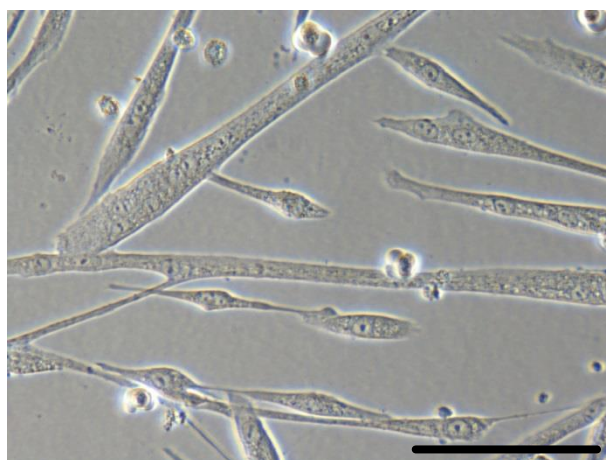**E**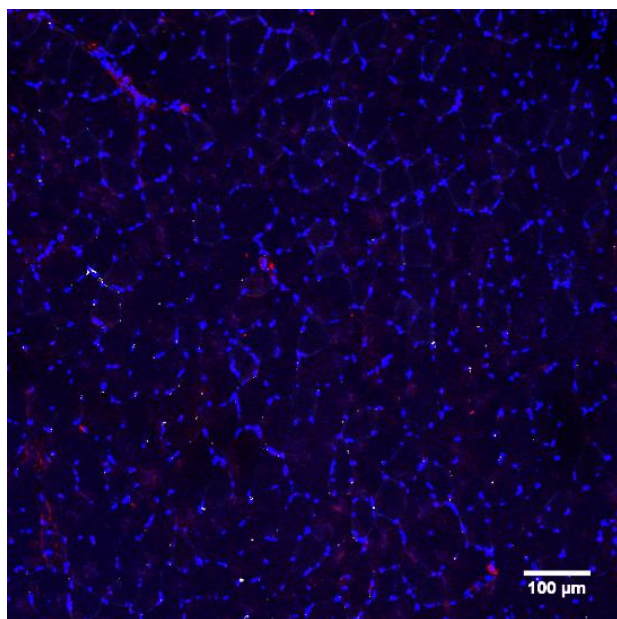

TdTomato DAPI

**F**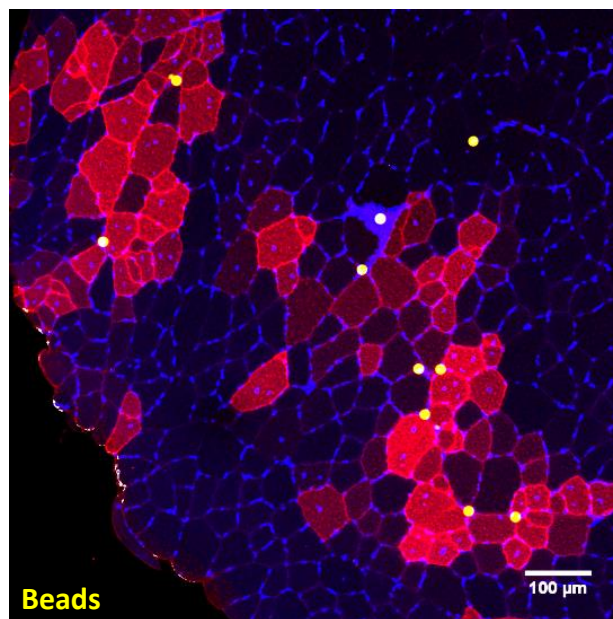

Beads  
TdTomato DAPI

Supplement: Supplementary file 4 — Additional file 4: S4 Fig. Characterization of murine MPC and MPC-SMC. (A) AChE and (B) CK activity was measured by enzyme kinetics and is represented by OD412nm and OD340nm at specific time points (AChE: 60 min, CK: 10 min). Enzyme activities were compared between skeletal muscle-derived mMPC and non-myogenic cells after 6 days in skeletal muscle differentiation medium. (C) Desmin expression of mMPC visualized by immunofluorescence staining. (D) Formation of multinucleated myotubes by mMPC during differentiation in skeletal muscle differentiation medium for six days in vitro was observed. TdTomato and nuclei were stained on histological cross sections of tibialis anterior muscles of (E) control untreated mice and (F) SHO mice 70 days after intramuscular injection with TdTomato mMPC and fluorescent beads. Scale bar = 100 μm. [file 13287_2020_1749_MOESM4_ESM.pdf]
