# Supplementary material for: Generation of myogenic progenitor cell-derived smooth muscle cells for sphincter regeneration
Source: Stem Cell Res Ther. 2020 Jun 12;11:233. doi: 10.1186/s13287-020-01749-w (PMC7291744; doi:10.1186/s13287-020-01749-w)
Supplement: Supplementary file 5 — Additional file 5: Supporting methods. [file 13287_2020_1749_MOESM5_ESM.docx]

**Supporting methods**

**Enzyme-linked immunocytochemistry**

For enzyme-linked immunocytochemistry, cells were detached from cell culture vessel surface by trypsinization and a cytospin was performed as following. 10 000 cells were suspended in 20-30 μl 1X PBS and pipetted into the lid of an. Eppendorf tube, who’s bottom was removed by using scissors before. The tube was carefully closed, attached to a cytospin stand above a glass slide and centrifuged at 1000*g for 7min. Then, the glass slide with the attached cells was kept at RT for at least 2 hours in order to let the cells dry. For immunocytochemical staining on cytospins, cells were washed once with 1X PBS. Then, 500 μl of the BD Cytofix/Cytoperm fixation (Becton Dickinson, NJ, USA) was used to cover cells and incubated at 4°C for 10 minutes for fixation. After washing the cells three times with 1X PBS, cells were covered with Ultavision Hydrogen Peroxide Block (Thermo Scientific, MA, USA) and incubated for 5 minutes at room temperature. After three additional washing steps, cells were covered with in 1X PBS 1:40 diluted anti-CD49a (MiltenyiBiotec GmbH, Bergisch Gladbach, Germany) or anti-CD146 antibodies (Thermo Scientific, MA, USA) diluted 1:100 in PBS and incubated at 37°C for 90 minutes. Cells were washed again with PBS and covered with ready-to-use biotinylated goat anti-rabbit or goat anti-mouse secondary antibodies (Thermo Scientific, MA, USA) and incubated for 60 minutes at 37°C. Afterwards, cells were washed with PBS, covered in 1:100 diluted horseradish peroxidase conjugated streptavidin (Vector Laboratories, Inc., CA, USA) and incubated for 30 minutes at 37°C. Subsequently, the cells were washed and incubated with Lab Vision™ Ready-To-Use AEC Substrate System (Thermo Scientific, MA, USA) for 10 minutes at RT. The reaction was stopped by washing with PBS three times and counter staining of nuclei was performed by covering the cells with Harris hematoxylin solution (Sigma-Aldrich Co. LLC, MO, USA) for 15 minutes at RT followed by thorough washing with tap water.

**Collagen gel lattice contraction**

Collagen gel lattice contraction was performed as previously described (54) with slight modifications. Briefly, 240 000 cells resuspended in 400μl DMEM/Ham’s F12 were mixed with 200 μl collagen I from bovine skin (Sigma-Aldrich Co. LLC, MO, USA) to reach a final concentration of 3 mg/ml collagen. The mixture was transferred to a well of a 24-well plate and the pH was adjusted to 7.2-7.4 by adding 3 μl 0.1M NaOH. After 30 minutes of incubation at 37°C, 5 % CO_2_ to allow gel solidification, 1 ml DMEM/Ham’s F12 was added and the gel was released from the walls of the 24-well plate to allow floating onto the medium. Images of the gels were acquired by light microscopy and % gel contraction was calculated by dividing the area of the collagen gel after 48 hours of incubation at 37°C with the initial gel area, each calculated applying ImageJ software. At least 3 gels were analyzed per cell type originating from a single muscle biopsy, and per cell type cells from three individual muscle biopsies were tested.
